# Birthing positions and mother`s satisfaction with childbirth: a cross-sectional study on the relevance of self determination

**DOI:** 10.1007/s00404-024-07770-1

**Published:** 2024-11-04

**Authors:** Nadine Scholten, Brigitte Strizek, Mi-Ran Okumu, Ibrahim Demirer, Jan Kössendrup, Lissa Haid-Schmallenberg, Malte Bäckmann, Arno Stöcker, Natalie Stevens, Anna Volkert

**Affiliations:** 1 Center for Health Communication and Health Services Research, Department for Psychosomatic Medicine and Psychotherapy, Faculty of Medicine, University Hospital Bonn, Venusberg-Campus 1, Gebäude 02, Auenbruggerhaus, 53127 Bonn, Germany; 2 Chair for Health Services Research, Faculty of Human Sciences and Faculty of Medicine and University Hospital Cologne, Institute of Medical Sociology, Health Services Research and Rehabilitation Science, University of Cologne, Eupener Str. 129, 50933 Cologne, Germany; 3 Department of Obstetrics and Prenatal Medicine, University Hospital Bonn, Venusberg Campus 1, 53127 Bonn, Germany; 4 Lake Cook Behavioral Health, 1718 Sherman Ave Suite 210, Evanston, IL 60202 USA

**Keywords:** Birthing position, Satisfaction with childbirth, Vaginal birth, Parturition, Birth setting, Labor

## Abstract

**Introduction:**

Considering the inconclusive evidence regarding the clinical benefits of specific birthing positions, emphasis has been placed on adhering to women’s preferences during the second stage of labour. Therefore, the present study aimed to assess the association between birthing position, the freedom to choose a birth position during the second stage of labour, and women’s subjective satisfaction with childbirth.

**Methods:**

We performed a cross-sectional survey of women 8 or 12 months after a vaginal birth in a hospital. The women were recruited via two cooperating health insurance companies. Multivariate analyses were conducted to assess the strength of the association between birthing position and maternal satisfaction with childbirth, with a particular focus on interactions with self-determination.

**Results:**

In total, the data from 761 women were analysed. The supine position was the most frequently reported birthing position in the second stage of labour at 77.5%. Notably, 39.0% and 30.5% of the women who gave birth in the dorsal and lateral supine positions, respectively, stated that the birth position was not chosen voluntarily. The regression models show a significant negative association between supine birthing position and satisfaction with childbirth, which is significantly related to self-determination. The most common reason for the adoption of a specific birthing position was instructions from medical staff.

**Discussion:**

The data provide insight into the perceived satisfaction with childbirth depending on the birthing position, whereby the relevance of self-determination is particularly evident. At the same time, self-determination is often not given, which is associated with reduced birth satisfaction.

## What does this study add to the clinical work

**Table:** 

The supine position was the most frequently reported birthing position in the second stage of labour, whereby over 30% stated that this birth position was not chosen voluntarily. Satisfaction with childbirth was significantly related to self-determination regarding the final birthing position.	

## Introduction

In Western countries, the supine position has historically prevailed as the most common birth position; it was promoted when instrumental delivery was introduced because it allowed unhindered access for obstetricians [13]. Formerly, upright, more physiological positions—for example, using birthing stools or standing upright—were more common in Western countries and in other cultures [13]. Thus, the introduction of the routine supine birth position is described as an intervention that was introduced without an evidence base [22]. To date, many studies have investigated how maternal position in the second stage labour affects the birth process, birth injuries, and infant outcomes [5, 20, 24]. Therefore, typically, a distinction is made between upright birth positions (kneeling, standing, or squatting) and supine birth positions (supine, lateral, or semi-recumbent with an elevated upper body) and the presence or absence of epidural anaesthesia. After considering 30 randomised controlled trials, a 2017 Cochrane review assessing the benefits of different birth positions for women without epidural anaesthesia came to the cautious conclusion that the upright birth position is preferable owing to a lower episiotomy rate, albeit with a slight tendency towards more first- and second-degree perineal tears; a lower rate of vaginal-operative births; and fewer signs of stress in the newborn [19]. Another Cochrane review investigating the benefits of various birth positions for women with epidural anaesthesia included eight randomised controlled trials [6, 12, 17, 23, 34, 38, 7, 36] and does not provide a clear recommendation; the findings suggest that although mothers reported higher satisfaction with the supine position, the position was associated with higher acid levels in the newborn [39]. It should be emphasized that the complete supine position was usually excluded in the studies [15, 19, 25] owing to the potential risk of aortocaval compression. A recent study on 2240 women concluded that an upright birthing position should be recommended regardless of epidural anaesthesia, as it was associated with fewer perineal injuries (episiotomy and perineal tears) [15]; furthermore, a pragmatic trial with 3093 nulliparous women with low-dose epidural anaesthesia endorsed the supine position [14].

To date, it remains unclear whether a specific birthing position is superior over the others; therefore, international guidelines [1, 27] repeatedly emphasize that women should adopt a birthing position that is most comfortable for them. Moreover, the NICE guidelines specify that lying flat on the back should be avoided [27], whereas the German guidelines state that women should be encouraged to adopt an upright position [1].

The cultural imprint and thus the conventional birth positions constitute one of many factors that influence a woman’s decision regarding which birth position to adopt [33]. In addition to the mother's wishes, the final birth position is influenced by the midwives and obstetricians present [26, 28, 31, 37, 40] and potential medical indications for a particular birth position.

Therefore, the primary aim of this study was to identify—from the mothers' perspective—external factors that led to the adoption of the final birth position and the extent to which external factors influencing a specific birth position in combination with the birthing position itself are associated with maternal childbirth satisfaction.

## Methods

The survey was conducted as part of the BMBF-funded MAM-Care study (FKZ: 01GY2110) from June to October 2023. To prevent a hospital-specific selection bias, a total of 4000 mothers were contacted by post through two cooperating health insurance companies and invited to voluntarily participate in an anonymous written survey. The study recruited women who gave birth to a live-born child in a hospital 8 or 12 months ago. This period was chosen to prevent potential re-traumatisation, and two-time points were chosen to control for possible COVID-specific restrictions. Only mothers who gave birth vaginally and without the use of instruments were considered. Mothers who had a primary or secondary caesarean section were excluded. The Ethics Committee of the Medical Faculty of the University of Cologne approved the study (22-1260). The data was collected anonymously, whereby the women's implicit consent was obtained by returning the questionnaire via post to the research institute. No personally identifying information was collected.

The final birthing position was surveyed, with the following response options: lying supine, lying on the side, standing/suspended (with a sling), kneeling/on all fours, standing/kneeling on one leg, squatting/using a birthing stool. We asked whether the participants had chosen the birthing position voluntarily (yes/no). If the birth position had not been chosen freely, the participants were asked to choose a reason from the following options: Mobility restricted by a CTG, mobility restricted by an epidural, instructions from medical staff, lack of knowledge about other birth positions. It was possible to give multiple answers and provide other reasons in free text.

Satisfaction with the course of the birth was assessed using the validated Satisfaction with Childbirth (SWCh) Scale [35]. All seven items were translated using the TRAP-D method [4] and assessed on a 7-point Likert scale ranging from 1 to 7 as in the original, with 7 depicting total satisfaction.

Other factors surveyed included the occurrence of perineal tears (first and second degree, third and fourth degree, do not know) and episiotomy (yes/no).

Descriptive analyses were performed by calculating mean values and standard deviations. Significant group differences were calculated. Multivariate linear regression models were used to examine the association between satisfaction with childbirth (SWCh) and supine birthing position (yes/no) (Models 1, 2, and 3) and explicitly for the factors that prevented free choice of birthing position (Model 4). Figures were generated with R, and the analyses were performed using Stata 18 and R.

Three linear regression models were fitted. Model 1 examined the relationship between maternal satisfaction with birth and the supine (lying supine, lying on the side) and upright (standing/suspended [with a sling], kneeling/on all fours, standing/kneeling on one leg, squatting/using a birthing stool) positions. In Model 2, we tested an interaction effect of choice X birth position. Maternal age (at the time of the survey), education (secondary school, academic secondary school or academic degree), and native language (German vs. non-German) were added to Model 2 as possible confounders, resulting in Model 3. Infant age at the time of the survey (8 vs. 12 months) had non-significant effects and was therefore not included as a confounder in the model. In Model 4, the association between specified reasons for lack of free choice of birth position and maternal satisfaction with birth was analysed. Robust standard errors were calculated to account for heteroscedasticity.

## Results

**Fig. 1:**
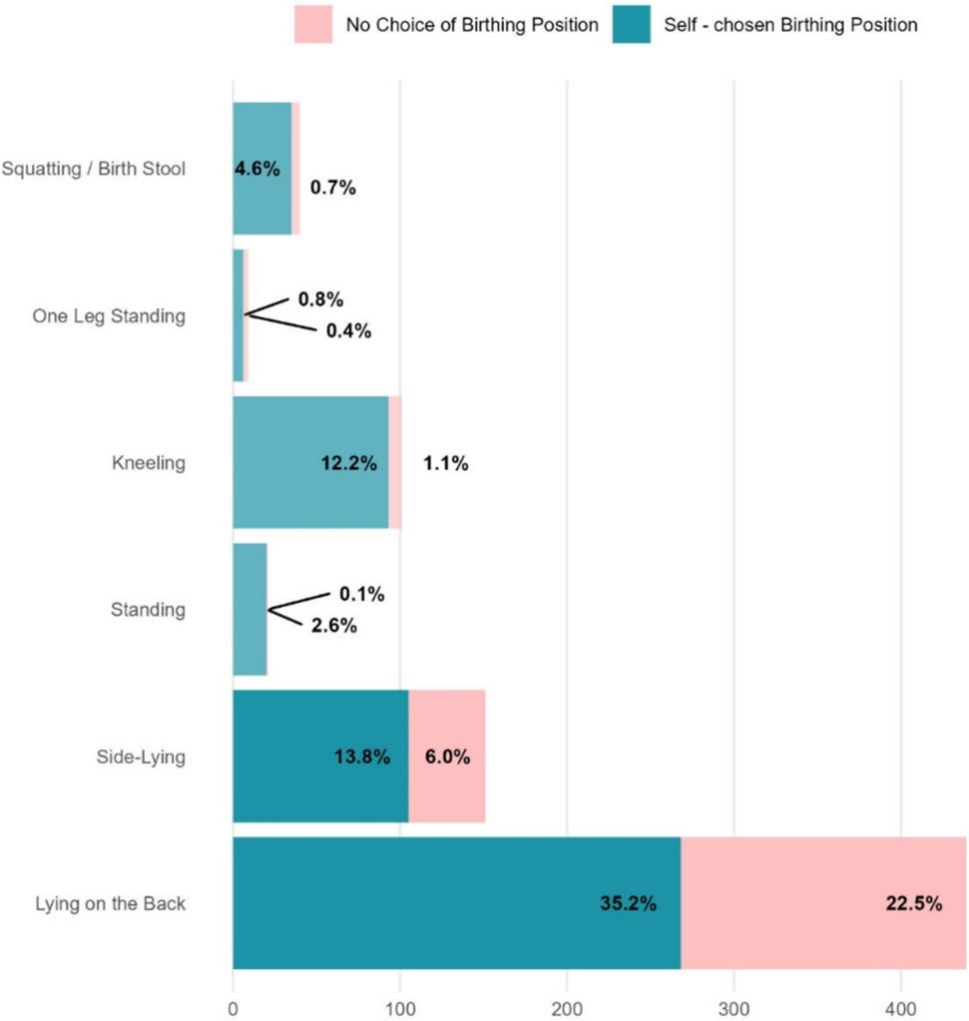
Distribution of birthing positions in the second stage of labour

We analysed 761 responses (complete cases) from 804 women who gave birth vaginally without the use of instruments. In total, 77.5% (*n* = 590) of women gave birth in the supine position, making it the most common birth position reported in this study (Fig. 1, Table 1). The supine position was further differentiated into the dorsal supine position (74.7%) and the lateral supine position (25.3%). Notably, 39.0% and 30.5% of women who gave birth in the dorsal supine position and lateral supine position, respectively, stated that the birth position was not chosen voluntarily. The standing position was the most common upright position (reported by 13.2%), followed by squatting (5.3%). With the exception of standing/kneeling on one leg (*n* = 9), all upright birthing positions were significantly more frequently self-selected than the supine positions (*p* < 0.01, Chi-square). Among women who determined the birth position themselves, there was a significant bivariate correlation between education and supine birth position (*p* < 0.01, Chi-square)—the proportion of women who gave birth to their child in a supine position decreased with increasing education (upright position: lowest educational level, 13.1%; highest educational level, 34.8%). The reason most frequently given by the women for not being able to choose freely was “instructions from medical staff” (*n* = 123; 54.2% of cases). “Restriction of mobility due to an epidural” (*n* = 59; 26.0%) and “restriction due to CTG” (*n* = 42; 18.5%) were mentioned much less frequently. The most common position assigned by staff was supine/lying on the back (73.1%), followed by supine/lying sideways (19.7%).

**Fig. 2:**
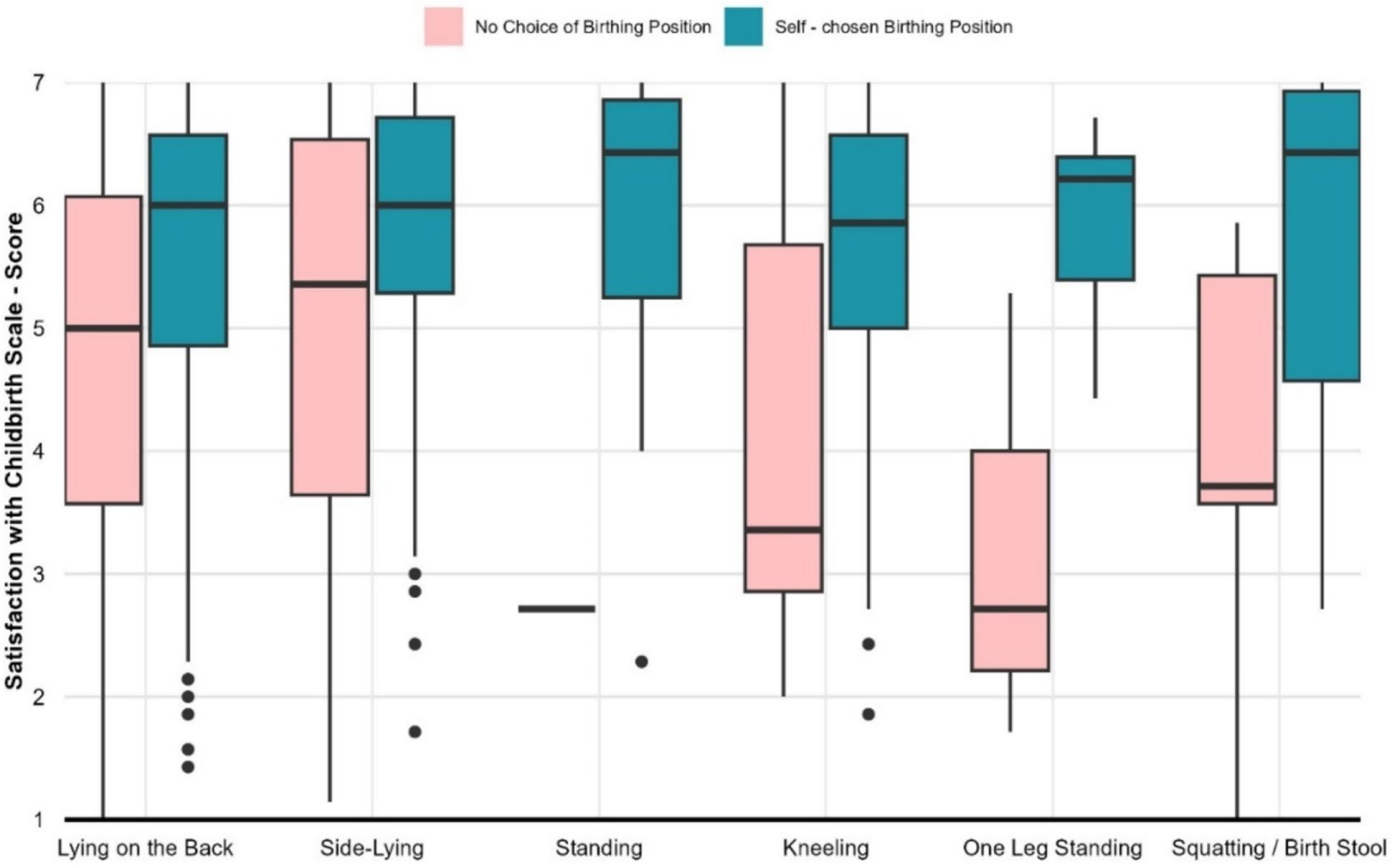
Maternal satisfaction with childbirth (SWCh) and birthing position

**Table 1:** Sample characteristics

Characteristic	*n* = 761^1^	Mean value SwCh	*p*-value^2^
Supine birthing position			0.1231
No	171 (22%)	5.503	
Yes	590 (78%)	5.344	
Birthing position			0.0351*
Lying on the back	439 (58%)	5.262	
Side-lying	151 (20%)	5.583	
Standing	21 (2.8%)	5.85	
Kneeling	101 (13%)	5.487	
One leg standing	9 (1.2%)	4.984	
Squatting/birth stool	40 (5.3%)	5.479	
Own choice of birthing position			0.0000
No	234 (31%)	4.72	
Yes	527 (69%)	5.672	
German as native language			0.0938
No	80 (11%)	5.209	
Yes	681 (89%)	5.34	
Education			0.5370*
Secondary school	114 (15%)	5.429	
Academic secondary school	195 (26%)	5.445	
Academic degree	452 (59%)	5.339	
Age	33.3 (4.0)	–	–

^1^Mean (SD); *n* (%)

^2^Wilcoxon rank-sum test

*Kruskal–Wallis test

Overall, there were significant differences in maternal satisfaction among birth positions (*p* = 0.04, k-Wallis). Moreover, there was a highly significant difference in maternal satisfaction depending on whether the birth position was self-selected or externally determined (*p* < 0.01, Wilcoxon rank-sum test test) (Fig. 2). The mean satisfaction rate for self-selected and externally determined birth positions was 5.67 (SD = 1.23) and 4.72 (SD = 1.65), respectively. When considering if the birth position was self-selected or externally determined, there was no significant difference between the birth positions themselves (k-Wallis: self-selected: *p* = 0.20, not self-selected *p* = 0.21).

**Table 2:** Linear regression models for the outcome of satisfaction with childbirth (SWCh)

	Model 1			Model 2			Model 3		
Characteristic	Beta	95% CI^1^	*p*-value	Beta	95% CI^1^	*p*-value	Beta	95% CI^1^	*p*-value
Supine position	− 0.16	− 0.40, 0.09	0.2	0.97	0.04, 1.9	0.040	0.97	0.04, 1.9	0.042
Own choice of birthing position				1.9	0.95, 2.8	< 0.001	1.9	0.95, 2.8	< 0.001
Supine position * own choice of birthing position				− 0.99	− 1.9, − 0.04	0.042	− 0.98	− 1.9, − 0.02	0.046
Education									
Academic secondary school							0.00	− 0.32, 0.31	> 0.9
Academic degree							− 0.04	− 0.32, 0.25	0.8
Age							0.00	− 0.03, 0.03	> 0.9
German as native language							0.32	0.01, 0.64	0.046
No. Obs	761			761			761		
R^2^	0.002			0.102			0.107		

^1^CI = Confidence interval

A negative but non-significant effect of the supine position on maternal satisfaction was observed (Model 1), and the relevance of freedom of choice regarding the birthing position was confirmed by the reversal of the effect in the subsequent models (Table 2). Overall, the significant effect of the supine position on satisfaction was evident across all subsequent models, with the effect being positive, but only when the voluntary nature of this position was considered. Freedom of choice of position also showed a consistently significant positive effect on satisfaction with childbirth. Moreover, the interaction effect of supine position and voluntariness was significant. Up to model 3, the explained variance increased to 10.72%. In this final model, women who were able to assume a birthing position voluntarily showed a 1.9 points higher mean birth satisfaction.

Among women who were not free to choose their birthing position, instruction from medical staff had the strongest negative and significant association (Coeff: − 0.56, *p* = 0.02). All other reasons for the lack of freedom to choose the birth position were not significantly associated with birth satisfaction (Table 3).

**Table 3:** Linear regression model for the outcome of satisfaction with childbirth (SWCh) considering only women with no choice of birthing position

Characteristic	Beta	95% CI^1^	*p*-value
No choice of birthing position			
Mobility limited by CTG	− 0.56	− 1.1, 0.02	0.060
Mobility limited by PDA	− 0.42	− 0.92, 0.08	0.10
Instructions from medical staff	− 0.56	− 1.0, − 0.10	0.017
Education			
Academic secondary school	0.34	− 0.48, 1.2	0.4
Academic degree	0.26	− 0.45, 0.97	0.5
Age	− 0.04	− 0.10, 0.01	0.14
German as native language	− 0.17	− 1.0, 0.69	0.7
No. Obs	227		
R^2^	0.052		

^1^CI = Confidence interval

**Fig. 3:**
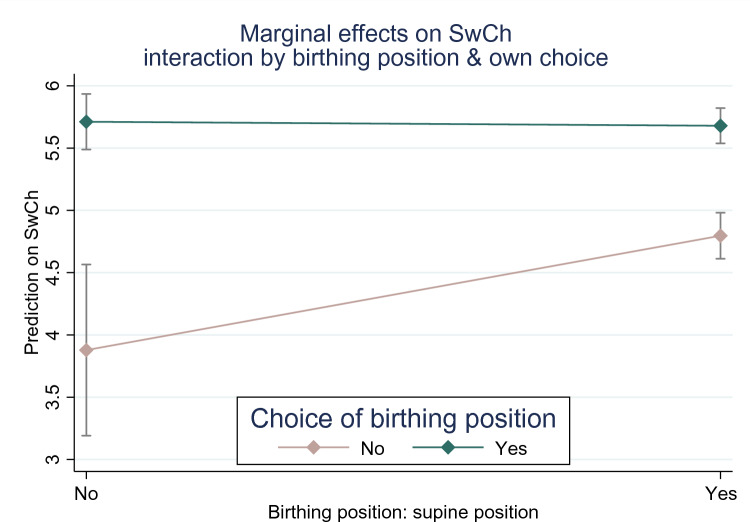
Marginal effects on satisfaction with childbirth interaction by birthing position and own personal choice

Figure 3 illustrates the marginal effects of birthing position and the possibility of own choice of birthing position on satisfaction with birth. The interaction reveals that when the birthing position is self-chosen (bluish-line), satisfaction with birth is high at a level around 5.8, irrespective of the position (supine or otherwise). Conversely, when the birthing position cannot be self-chosen (flesh-coloured line) and no supine position was taken (left-side on X-axis) satisfaction is substantially lower. However, this decrease in satisfaction with birth can be counteracted when having a supine position at birth (right side on the X-axis).

## Discussion

There is currently a trend towards upright and thus more physiological birthing positions; however, owing to the weak evidence regarding clinical outcomes, most guidelines prioritize the wishes of the woman giving birth [1, 27]. The present analyses demonstrate that the birth position itself had no significant influence on maternal satisfaction with childbirth but that the voluntariness of the birth position was strongly correlated with birth satisfaction. The most frequently reported birth position was dorsal and lateral supine position (77.5%). This frequency of the supine position is consistent with international data, with 68% of U.S. mothers surveyed stating that they had given birth to their child in the supine position [9]; similar figures have been reported in Sweden (83.9%) [18] and Brazil (98.3%; left lateral and 45° reclined) [8]. Overall, however, there are limited data available from observational studies [11] to obtain an up-to-date international comparison of birthing positions.

In principle, at least one study describes a higher level of satisfaction with the supine position compared to the upright birthing position [6]. At the same time Fig. 3 highlights that having a supine position has a negative association with birth satisfaction, but only when not being able to choose the birthing position.

The present study revealed that the supine birthing position was not actively chosen by approximately 37% of the women. The most common reason cited was instructions from medical staff. In addition to obstetric interventions that make a supine position necessary, other possible causes that favour the prioritisation of the supine position have been mentioned in previous studies, particularly with a focus on midwives [26]. These include the midwives' experience with upright birth positions, for example, through their training and the routines and skills developed as a result [16, 22].

Particularly noteworthy is the high number of women who did not choose the birth position themselves and an associated lower level of satisfaction with birth. Despite the relevance of shared decision-making in the field of obstetrics [3, 10, 29, 32], there still seems to be room for improvement, at least in terms of birth position. As the benefits of continuous CTG monitoring in low-risk births are generally controversial, its influence on the choice of birthing position must be critically reviewed [2]. Regarding the potential influencing factors of the final birth position, Priddis et al. showed a possible association with the educational level of the women giving birth, where more highly educated women gave birth in a supine position less frequently [30]. This is consistent with our data, where women with a higher level of education gave birth in an upright position more often.

Nevertheless, it remains debated whether it is sufficient to offer women the choice of birthing position in a situation where strong social conventions facilitate the adoption of a supine position [21].

## Study limitations

Recruitment via health insurance companies achieved a moderate response rate of 27.6%. Because it was not necessary to approach individual hospitals, recruitment without provider bias was possible. However, because of this approach, no information on the birth clinic was available. The low number of women with a migration background and lower level of education in our data suggests that this group was less well reached by the survey. Owing to the focus on self-reported data, social desirability bias cannot be excluded. Only the women's self-reports were available for analysis, and therefore the objective reasons for the staff's instructions to adopt a specific birthing position remain unknown.

## Conclusion

The results highlight the relevance of choosing the birth position voluntarily, as it was significantly associated with maternal satisfaction with the birth experience. Nevertheless, many women reported that the final birth position had not been chosen by themselves, with instructions from medical staff being the most common reason cited. To increase the subjective satisfaction of women with their birth experience, measures must be taken to enable them to adopt a preferred position during the second stage of labour. The first step includes training and further education of medical staff and the empowerment of women to understand and better communicate their preferences.

## Data Availability

The datasets used and/or analysed during the current study are available from the corresponding author on reasonable request.
